# Photonics and microwaves merge to improve computing flexibility

**DOI:** 10.1038/s41377-025-01933-8

**Published:** 2025-09-04

**Authors:** Hongwei Wang, Guangwei Hu

**Affiliations:** School of Electrical and Electronic Engineering, 50 Nanyang Avenue, Nanyang Technological University, Singapore, 639798 Singapore

**Keywords:** Optical techniques, Other photonics

## Abstract

In artificial neural networks, data structures usually exist in the form of vectors, matrices, or higher-dimensional tensors. However, traditional electronic computing architectures are limited by the bottleneck of separation of storage and computing, making it difficult to efficiently handle large-scale tensor operations. The research team has developed a photonic tensor processing unit based on a single microring resonator, which performs tensor convolution operations in multiple dimensions of time, wavelength, and microwave frequency by precisely adjusting the operating state of multi-wavelength lasers. This innovative design increases the photonic computing density to 34.04 TOPS/mm², significantly surpassing the performance level of existing photonic computing chips.

The rapid development of artificial neural networks and deep learning has led to an increased demand for computing power and efficiency in modern computational tasks^1,2^. However, traditional silicon-based electronic computing is constrained by Moore’s law, which hinders its ability to scale up and improve efficiency^3^. Photonic computing, on the other hand, is a novel technology that offers high processing speeds and high energy efficiency, attracting growing interest^4–12^. Recently, a research team from the Institute of Semiconductors of the Chinese Academy of Sciences proposed a highly integrated photonic tensor core that uses high-dimensional light waves and microwave multi-domain multiplexing technology to significantly increase computing density and promote photonic computing chips towards high performance. The research results were published in the latest issue of the journal Light: Science & Applications^13^.

## Highly integrated photonic tensor core: a single device for multi-dimensional computing

Most traditional photonic computing chips use a micro-ring array architecture based on the thermo-optical effect, which has limited computing density and reduced accuracy due to thermal crosstalk. The new architecture^13^ proposed in this study abandons traditional thermo-optical control and uses microwave-lightwave hybrid multiplexing technology to map data storage and computing to different physical dimensions, greatly improving information processing capabilities. This method uses a micro-ring resonator as the unit core and controls the wavelength of light waves to achieve high-precision weight adjustment and reduce chip area. On the basis of traditional wavelength multiplexing (WDM) technology, they additionally introduced microwave frequency as an information dimension, expanded matrix operations to tensor calculations, and improved the flexibility of data input channels. Through multi-channel low-speed processing of microwave signals, the dependence on high-power high-speed analog-to-digital converters (DA/AD) is reduced, further optimizing system power consumption. The research team used this photonic tensor core to conduct experiments on handwritten digit recognition (MNIST dataset). The experimental results show that the recognition accuracy of the convolutional neural network (CNN) model based on photonic computing on the test set is as high as 96.41%, close to the theoretical prediction of 96.79%. In addition, the chip computing speed reached 245.33 GOPS, demonstrating powerful information processing capabilities.

## The future of photonic computing

**Fig. 1:**
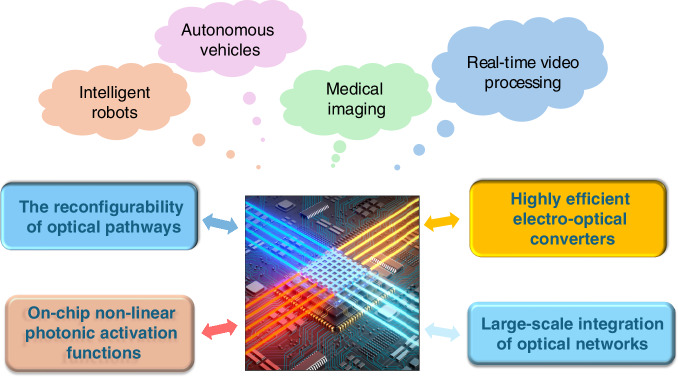
Schematic view of the challenges and applications of photonic computing

This breakthrough research not only improves the performance of photonic computing chips, but also provides new possibilities for future applications such as biometric recognition, telemedicine, and edge AI computing. As claimed, this architecture can further improve parallel computing capabilities by increasing the number of wavelengths and microwave multiplexing channels, laying the foundation for the commercialization of photonic computing chips. However, as shown in Fig. 1, photonic computing still faces a number of challenges at present, including:

i. Optimize the manufacturing process of optical components to overcome the significant insertion loss and phase error accumulation associated with large-scale integrated optical circuits. This will facilitate the expansion of computational network layers to enhance the capability of optical computing in handling complex tasks.
ii. Improve the reconfigurability of optical circuits to expand the computational infrastructure of photonic computing, enabling adaptability to a wider range of computational tasks.
iii. Introduce on-chip nonlinear photonic activation technologies to extend the depth of photonic network computing and integrate photonic computing with existing electronic computing technologies, creating a comprehensive computational ecosystem.
iv. Enhance the efficiency, speed, and sensitivity of optoelectronic converters to improve the overall energy efficiency of optical computing.

Future research will focus on addressing these challenges to develop hybrid photonic computing architectures, explore new optical materials, and build end-to-end photonic computing application platforms. With the continued advancement of photonic computing in photonic integrated circuits and optical neural networks, photonic computing will be applied to a wider range of real-world scenarios, from intelligent robots and autonomous vehicles to medical imaging and real-time video processing. Additionally, photonic multidimensional computational capabilities will emerge as a solution to traditional electronic computing challenges, potentially driving the advancement of artificial intelligence and machine vision to new stages.
